# Effect of Fluorane Microcapsule Content on Properties of Thermochroic Waterborne Topcoat on *Tilia europaea*

**DOI:** 10.3390/polym14173638

**Published:** 2022-09-02

**Authors:** Zixin Yang, Yan Han, Wenwen Peng, Lin Wang, Xiaoxing Yan

**Affiliations:** 1 Co-Innovation Center of Efficient Processing and Utilization of Forest Resources, Nanjing Forestry University, Nanjing 210037, China; 2 College of Furnishings and Industrial Design, Nanjing Forestry University, Nanjing 210037, China

**Keywords:** fluorane microcapsules, waterborne topcoat, optical properties, mechanical properties, self-healing performance

## Abstract

In a particular temperature range, 1, 2-benzo-6-diethylamino-fluorane microcapsules (fluorane microcapsules) exhibit a good color-changing function. For the coating on wood surfaces, embedding fluorane microcapsules, good weather resistance, light retention, color retention, impact resistance, and wear resistance are essential. However, the effect of fluorane microcapsule content on its properties has not been verified. Therefore, in this paper, the orthogonal test is designed with the fluorane microcapsule content, drying temperature, and drying time as test factors to identify the most influential factors. Then, by embedding microcapsules into the waterborne coating on wood substrates, the performance of the waterborne topcoat was investigated. The results show that the color of the waterborne topcoat with fluorane microcapsules on a basswood (*Tilia europaea*) surface can change between yellow and colorless when the temperature rises and falls, achieving reversible thermochromism. The activation temperature was 32 °C, and the range of discoloration temperature was 30–32 °C. The topcoat with a 15% fluorane microcapsule content had the best comprehensive performance. The color difference was 71.9 at 32 °C, the gloss was 3.9% at 60°, the adhesion grade was 0, the hardness was 2H, the impact resistance was 10 kg·cm, the elongation at the break was 15.56%, and liquid resistance was outstanding. After aging tests, the color difference of the topcoat with 15% fluorane microcapsules was more obvious. The damaged area of the topcoat with the addition of 15% fluorane microcapsules was smaller, indicating it had a better aging resistance. The experimental results lay the foundation for the preparation of intelligence-indicating and decorative waterborne coating.

## 1. Introduction

Wood, as a natural material, has a particular structure and color [1,2,3,4,5,6] that often makes it applicable to the field of construction and to furniture [7,8,9]. The wood products’ surfaces are typically covered with multiple layers of coatings. The topcoat is typically the layer that is most impacted by solicitations from outside, such as UV light, moisture, and chemicals [10]. Compared with the traditional solvent-based topcoat, the water-based topcoat eliminates the fire hazards in construction, reduces air pollution, and saves a lot of money [11,12,13].

Based on a temperature difference, thermochromic microcapsules can spontaneously change their color. Among the organic compounds as colorants, the advantages of fluorescent agents are low discoloration temperatures and high sensitivity, meeting the discoloration requirements of wood between yellow and colorless [14]. The application of microcapsules in many fields is relatively mature, and there is much research on color-changing microcapsules, and even relatively mature experimental results have been obtained. Gao et al. [15] described an innovative approach for the in situ synthesis of a fluorescent carbon dot and its assembly in polyelectrolyte microcapsules, obtaining the highly biocompatible nanocomposed microcapsules with excellent luminous properties that help create in vitro images and the ability to recognize them. In order to enable optical imaging of the capsules in a waterborne solution, Yashchenok et al. [16] sought to immobilize active ingredients such as fluorescent dyes, quantum dots, and metal nanoparticles in polymeric shells. Campana et al. [17] explored the design, fabrication, and characterization of a low-cost microsystem for mushroom-based paint with cs43 immersed in the algal microencapsulation substrate. Multiphysics simulations were used to illustrate the behavior of the fluid inside the device and to evaluate the size of the final capsule. Di Girolamo et al. [18] studied the development of a high-flow filtration system based on a new microcapsule of water gel, a selective filter polymer. The results of the construction were obtained by the injection of fragmentation and layering techniques of a hollow polyelectrolyte envelope with a selective permeability gel glucate microcapsule.

With the improvement of quality in life, people have higher requirements for the appearance of furniture, and the changeable color of furniture has also become a means to attract customers. A thermochromic topcoat can be achieved by embedding thermochromic microcapsules into topcoats [19,20,21]. When this type of coating is applied on the surface of wooden furniture, its surface color can alter when the surrounding temperature changes. Wood surface finish is required to have good weather resistance, light retention, color retention, impact resistance, and wear resistance. However, there is no in-depth study on the color-changing microcapsules applied to the water-based finish on the surface of the wood.

In previous research [22], the performance of wood waterborne primer (another important layer in the multiple layers of coatings besides the topcoat) with thermochromic fluorane microcapsules was recorded. In this paper, thermochromic fluorane microcapsules were added to the waterborne topcoat. First, the factor significantly affecting the color-changing effect is determined through an orthogonal test with thermochromic fluorane microcapsule content, drying temperature, and drying time as variables, and then the optimal content of fluorane microcapsules, which can achieve a better color-changing effect, gloss, and liquid-resistance of the topcoat, was studied. To find out how long the topcoat with fluorane microcapsules will be effectively thermochromic, the artificially accelerated aging property, which included dry-heat aging and ultraviolet (UV) photooxidation, was discussed. This study’s findings serve as a technical reference for wood surfaces coated with waterborne and thermochromic topcoats.

## 2. Materials and Methods

### 2.1. Preparation of Color Changing Finish Topcoat

The orthogonal test is designed with the 1, 2-benzo-6-diethylamino fluorane microcapsule (Shenzhen Oriental Color Change Technology Co., Ltd., Shenzhen, China) content, drying temperature, and drying time as test factors, as shown in Table 1. Samples 1#–4# in Table 2 correspond to the orthogonal test. The total mass of every waterborne thermo-chromic topcoat sample was 4.00 g. Samples 5#–10# in Table 2 were used for an optimization test based on the orthogonal test.

Take the preparation of sample 1# as an example: the 0.6 g fluorane microcapsules and 3.4 g clear waterborne topcoat (Nippon Paint Co., Ltd., Shanghai, China), which contained waterborne acrylic copolymer dispersion, a matting agent, an additive, and water, with a solid content of 30%, were weighed, mixed, and coated on the basswood substrates (*Tilia europaea*, Suqian Qingyun Wood Industry Co., Ltd., Suqian, China) by an SZQ four-sided topcoat preparer (Chengdu Zhentong Trade Co., Ltd., Chengdu, China) to obtain a waterborne film with a thickness of 60 μm. The coated samples were moved to a 35 °C DHG-9643BS-Ⅲ electric-heating constant-temperature blast-drying oven (Shanghai Xinmiao Medical Instrument Co., Ltd., Shanghai, China) and heated for 20 min to dry. Then they were taken out and naturally cooled to room temperature. Following gentle polishing with 800 fine sandpapers, the powder was removed with a dry cloth. Two coats of topcoats were applied. The coated samples were then transferred to the oven for 2 h at 35 °C to dry. Following natural cooling, a dry topcoat was made as sample 1#. Other samples were prepared using the same technique as sample 1#.

### 2.2. Testing and Characterization

The color difference was tested using a SEGT-J portable chromatic difference instrument (Zhuhai Tianchuang Instrument Co., Ltd., Zhuhai, China) [23]. The color difference value (∆*E*_94_) was calculated according to Formula 1:

∆*E*_94_ = [(∆L*)^2^ + (∆a*)^2^ + (∆b*)^2^]^1/2^ (1)

In Formula 1, ∆*L* = L* − L*’, ∆*a* = a* − a*’, ∆*b* = b* − b*’. L*, a*, and b* represented chromatic parameters of original topcoats that had not undergone any aging or liquid resistance testing. L* represents the lightness of the color, the value +a* represents redness, the value −a* represents greenness, the value +b* represents yellowness, and the value −b* represents blueness. L*’, a*’, and b*’ represented the chromatic parameters of the treated topcoats through aging tests or liquid resistance tests.

An LS191 intelligent gloss meter (Shenzhen Linshang Technology Co., Ltd., Shenzhen, China) was used to measure topcoats’ gloss [24]. A pencil hardness tester (Beijing Wowei Technology Co., Ltd., Beijing, China) was used to measure the topcoats’ hardness [25]. An F107 topcoat scriber (Beijing Times Jiaxiang Technology Co., Ltd., Beijing, China) was used to measure the topcoats’ adherence. A film impactor tester (Dongguan Daxian Automation Equipment Co., Ltd., Dongguan, China) was used to assess the impact resistance of the topcoats [26]. The universal mechanical testing equipment (Shandong Kaifeng Testing Technology Co., Ltd., Jinan, China) was used to measure the elongation at the break of the topcoats.

Using a 15% NaCl (Hebei Langfang Nabo Chemical Technology Co., Ltd., Langfang, China) solution, 70% medical ethanol (Hebei Langfang Nabo Chemical Technology Co., Ltd., Langfang, China), detergent (25% fatty alcohol ethylene oxide and 75% water, Shanghai Hutchison Whitecat Co., Ltd., Shanghai, China), and red ink (Shanghai Fine Cultural Articles Co., Ltd., Shanghai, China), the topcoats’ performances for resistance to liquids were assessed [27]. After a filter paper was submerged for 30 s in the test solution, it was picked up with a tweezer and placed onto the test area. After wiping away the test solution, a toughened glass cover was immediately placed over the sample. After 24 h, the filter paper and the toughened glass cover were moved away. The remaining liquid on the topcoats’ surfaces was absorbed using absorbent paper. Finally, the topcoats were examined on color difference and gloss. The classification of the liquid resistance of the topcoats is shown in Table 3.

A DHG-9643BS-Ⅲ electric-heating constant-temperature blast-drying oven (Shanghai Xinmiao Medical Instrument Co., Ltd., Shanghai, China) was used to carry out the dry-heat aging of artificially accelerated aging tests at 120 °C and 160 °C for 0–40 h, respectively. An ultraviolet weather resistance test chamber (Nanjing Environmental Testing Equipment Co., Ltd., Nanjing, China) was used to conduct the UV photooxidation of artificially accelerated aging tests [28].

The microstructure and chemical composition of the topcoats were examined by a Zeiss Axio Scope A1 optical microscope (OM, Carl Zeiss AG, Oberkochen, Germany), a Quanta-200 scanning electron microscope (SEM, FEI Co., Ltd., Hillsboro, OR, USA), and an FTIR-850 infrared spectrum analyzer (FTIR, in the form of pellets prepared with KBr, Tianjin Gangdong Science and Technology Development Co., Ltd., Tianjin, China), respectively.

All tests were repeated 4 times with an error of less than 5%.

## 3. Results and Discussion

### 3.1. Orthogonal Experiment

**Figure 1:**
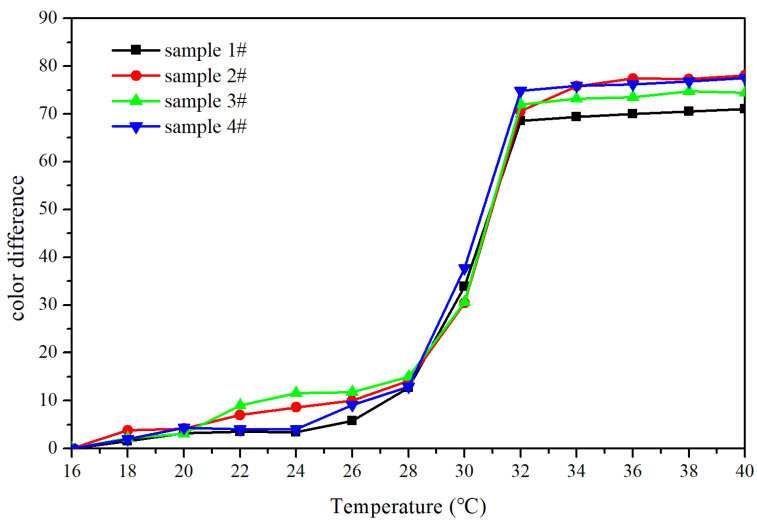
The color difference of topcoats with fluorane microcapsules under different temperatures.

An orthogonal experiment of three factors and two levels was used to determine the variables that affect the performance of the topcoat. As shown in Figure 1, when the temperature rose to 28 °C, the color difference of the topcoat changes significantly. When the temperature rose from 28 °C to 30 °C, the color difference changed and presented a slow upward trend with a small amplitude. The activation temperature, which means the temperature when the color difference of the topcoat essentially attained its maximal value and had been in a steady state, was 32 °C.

Table 4 shows the different color results during the heating process (16–32 °C) and the range of the orthogonal experiment. As can be seen from Mean 1 and Mean 2, the drying temperature and drying time had the most impact on the topcoat color difference. Therefore, the dry temperature of 60 °C and drying time of 2 h were fixed, and the fluorane microcapsule content was modified. The effect of the fluorane microcapsule content (5%, 10%, 15%, 20%, 25%, 30%) on the properties of the topcoat was examined.

### 3.2. Optical Properties

**Figure 2:**
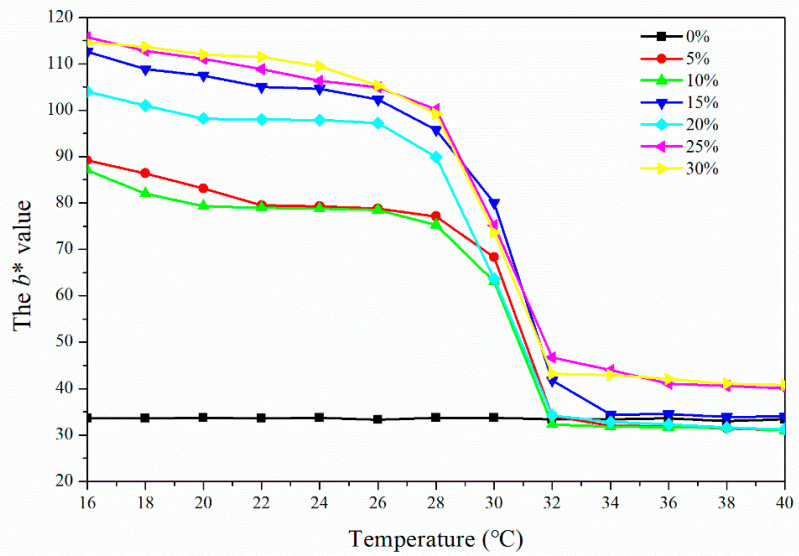
The effect of the heating process (16–40 °C) on the b* value of the topcoat with different contents of fluorane microcapsules.

As can be seen from Figure 2, the value of the colorimetry parameter b* of the topcoat with 0% fluorane microcapsules was kept at 33.0–34.0 with an increasing test temperature, which showed that the topcoat’s color changed less. For other topcoats with fluorane microcapsules, the reduction of the b* value indicates that the color of the topcoat progressively changes from yellow to colorless. During 16–28 °C, the b* value did not obviously change. The b* value started a downward trend at 30 °C, changed significantly when the temperature rose to 32 °C, and then tended to be stable at 32–40 °C. Thus, the topcoat changed from yellow to colorless at 32 °C, which was the activation temperature of these samples.

**Figure 3:**
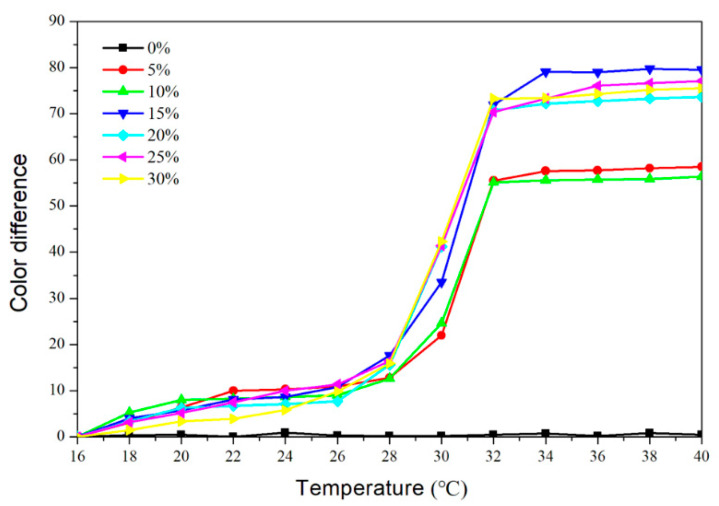
The color difference of topcoats with fluorane microcapsules during the heating process (16–40 °C).

**Figure 4:**
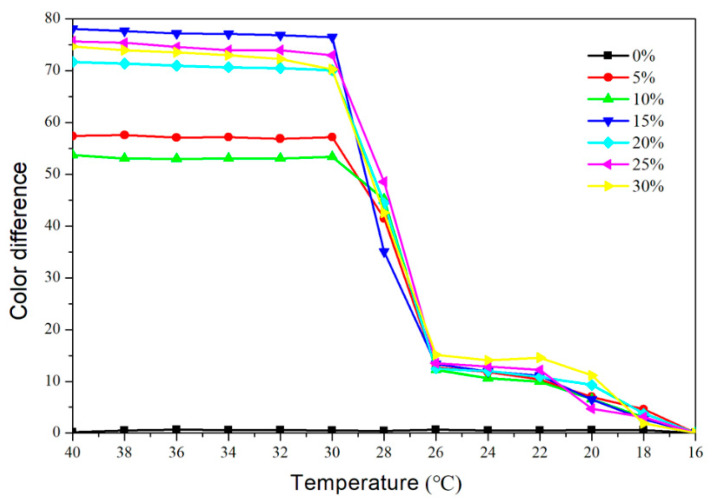
The color difference of topcoats with fluorane microcapsules during the cooling process (40–16 °C).

Figure 3 and Figure 4, respectively, depict the trend of the topcoat’s color difference as it warmed up to 40 °C and then cooled down to 16 °C. The color difference of the topcoat at 16–28 °C was essentially below 20 when the microcapsule content was 5–30%. The color difference essentially achieved its maximum value at 32 °C. The topcoat without the fluorane microcapsules had a color difference between 0 and 1.0, meaning it had little color-changing effects. Figure 3 and Figure 4 demonstrate that the color difference of the topcoat increased significantly in the range of 30–32 °C and presented a stable maximum value in the range of 32–40 °C. When the added content was greater than or equal to 15%, the topcoats showed a better color-changing effect. The color difference of the topcoat containing 15% microcapsules increased to 71.9 when the temperature reached 32 °C.

At the test temperature of 32 °C, the color changes from yellow to colorless, revealing the wood’s original color. The color of the topcoat changed back to the original yellow when the temperature dropped further. The color value of the topcoat with fluorane microcapsules was consistent with that of the primer with the fluorane microcapsules [22], showing that the addition of the microcapsules did not influence the temperature range at which the topcoat changes color and that reversible thermochromic color change was still achievable.

Table 5 demonstrates that the addition of fluorane microcapsules significantly affects the gloss of the coating’s surface. When the number of fluoride microcapsules in the coating is fixed, the brightness of the topcoat increases with an increasing angle of incidence. When at an angle of 60°, the gloss of topcoats without fluorane microcapsules was 33.2%. For samples 6#–11#, at the same angle of incidence (60°), the gloss of topcoats with 5% fluorane microcapsule content is the highest. When the microcapsule content exceeded 5%, the gloss of the topcoat reduced as the content did too. This could be because the content of microcapsules increased to some extent so that the topcoat’s surface roughness increased, resulting in the scattering of light and a decrease in the gloss of the topcoat [29,30,31]. The results show that the gloss of the topcoats is higher when the contents are between 5% and 15%.

### 3.3. Mechanical Property

Table 6 displays how microcapsules affect a topcoat’s hardness, impact resistance, adhesion, and elongation at break. According to the test, the increase in microcapsule content improved impact resistance. The hardness rose from H to 3H. The adhesion grade of the topcoat was 0 when the microcapsule content ranged from 0 to 20%. The adhesion dropped to grade 1 when the content was more than 20%. The topcoat containing 0–30% microcapsules had an increase in impact resistance from 5 kg·cm to 11.0 kg·cm. The trend of the elongation at the break was arched. The maximum elongation at the break of the topcoat was 23.46% when the content of the topcoat was 10%. The results indicate that fluorane microcapsules added to the topcoat can enhance its mechanical and physical properties. The microcapsules made the topcoat more fragile and lowered the topcoat’s mechanical qualities as the content increased.

### 3.4. Liquid Resistance

According to Table 7, the color difference determined from the LAB colorimetric parameters was measured before and after the tests of resistance to liquids. The color difference of topcoats treated with various test solutions mainly exhibits an upward trend with the rise in microcapsule content. The greater the color difference of the topcoat, the lower its resistance to liquids. Following the testing with four resistant liquid solutions, the red ink’s performance on the color difference was more obvious. Table 8 indicates the gloss of topcoats before and after tests of resistance to liquids. After the liquid resistance test for four test solutions, the change in the gloss of topcoats was not significant. Table 9 shows how the coatings’ resistance to liquid levels varied. The lower the resistance level, the better the liquid resistance. The coatings that contain 0 to 30% microcapsules have grade 1 of resistance to NaCl and ethanol. The resistance of the coatings to the detergent was better, which was grade 1 when the content of the microcapsules was 0–15%. When the content of the microcapsules was higher than 15%, the resistance of the detergent liquid dropped to level 2 with a slight imprint of discontinuous compression. However, as the content of the microcapsules increased, the resistance of the topcoat to the red ink decreased. These results show that the topcoat was less resistant to red ink than the other three kinds of liquid. This is probably due to the degree of solidification of the coating itself and its formation. The coatings which contain the fluorane microcapsules were less resistant to red ink. This is because the coating was initially yellow at room temperature and upon examination with red ink. Under the capillary action, the color changed from yellow to red with a greater difference in color and a larger imprint [32,33,34,35,36,37].

### 3.5. Microstructure and Infrared Spectroscopy Analysis

**Figure 5:**
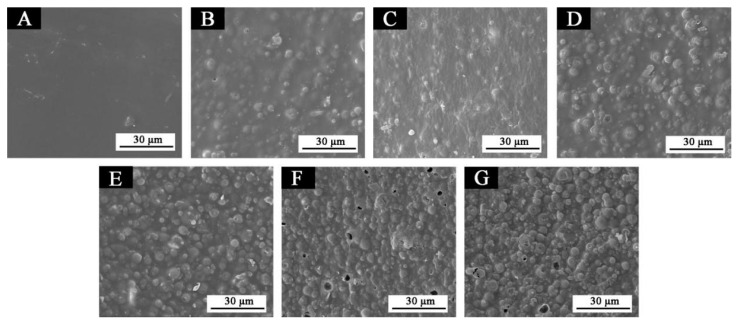
SEM of topcoats with different concentrations of fluorane microcapsules: (**A**) 0%, (**B**) 5%, (**C**) 10%, (**D**) 15%, (**E**) 20%, (**F**) 25%, (**G**) 30%.

The micromorphology of a waterborne topcoat on basswood with various fluorane microcapsule contents is shown in Figure 5. The fluorane microcapsule content was continuously added, which caused the shape of the particles on the topcoat’s surface to become more and more visible. There were minor holes on the topcoat’s surface when the content was between 25% and 30%, which may have been created by adding too much content.

**Figure 6:**
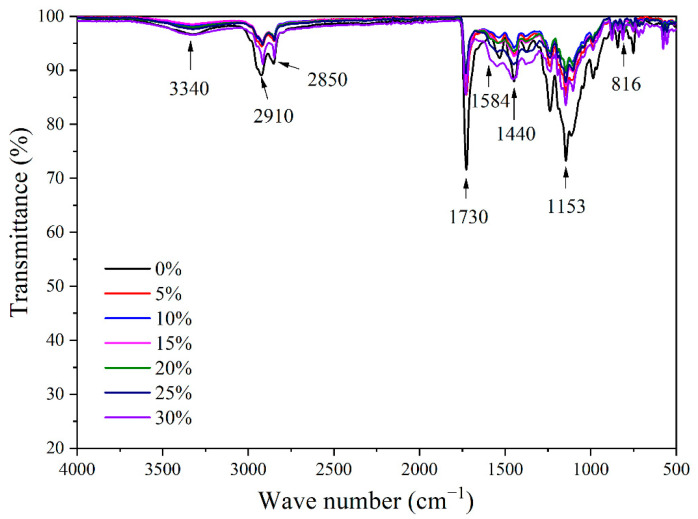
FTIR of topcoats containing different concentrations of fluorane microcapsules.

The FTIR spectra of the coatings with various fluorane microcapsule contents are shown in Figure 6. At 3340 cm^−1^, the tensile vibration absorption of -NH and -OH were superimposed. C-O-C absorbed stretching vibrations at 1153 cm^−1^, and CH_2_ had a stretching vibration peak at 1440 cm^−1^. A strong carbonyl characteristic absorption was present at 1730 cm^−1^, while the tensile vibration of -CH_3_ was present at 2910 cm^−1^. The triazine ring’s peak for both stretching and bending vibration absorption was found to be at 1584 cm^−1^ and 816 cm^−1^, respectively. No peak disappeared or appeared when fluorane microcapsules were added to the topcoat. The thermochromic effect can still occur when fluorane microcapsules are added to the basswood surface waterborne topcoat layer, proving that there was no chemical reaction between fluorane microcapsules and the topcoat on the wood’s surface.

When 1, 2-benzo-6-diethylaminofluorane reacts with an acid chromogenic agent, which results in an electron transfer, the lactone ring of the compound opens and forms a conjugated chromogenic structure when the temperature is lower than the solvent’s melting point [38,39]. The microcapsules turn yellow when the central carbon atom transitions from an sp^3^ etherified state to an sp^2^ hybrid plane configuration. The solvent turns into a liquid as the system’s temperature rises, and as a result, it separates from the 1, 2-benzo compound. The conjugal structure of quinone achieves a reversible transformation into a lactone ring structure in the liquid state [22].

### 3.6. Artificially Accelerated Aging Experiment

**Figure 7:**
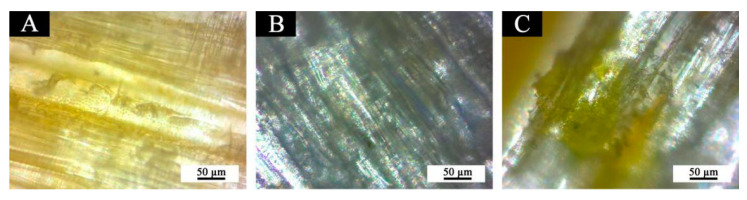
OM: (**A**) basswood, (**B**) basswood coated with a topcoat without microcapsules, (**C**) basswood coated with a topcoat containing 15% fluorane microcapsules.

In order to further explore the influence of the distribution of microcapsules in wood and coating on the thermochromic effect, the uncoated and coated basswood with and without 15% fluorane microcapsules, performs well across the board in terms of color contrast, gloss, liquid resistance, and micromorphology, was observed under an optical microscope, as shown in Figure 7. Figure 7A shows the microscopic structure of basswood slices observed under an optical microscope, Figure 7B shows the basswood slices coated with a waterborne topcoat and Figure 7C shows the basswood slices coated with a waterborne topcoat with 15% fluorane microcapsules. Because fluorane microcapsules were yellow at room temperature, the particle size was very small. Figure 7C shows that the waterborne topcoat on the basswood surface was yellow, and the microcapsules were well dispersed without a large agglomeration phenomenon.

Waterborne topcoats with and without 15% fluorane microcapsules on the basswood surface were divided into three equal pieces and labeled as three groups of blank samples. The topcoat was subjected to an artificially accelerated aging experiment in three different environments: an oven at 120 °C, an oven at 160 °C, and a UV climate resistance test room. The topcoat’s surface condition was monitored during the experiment. Table 10 illustrates how the aging process affected the color difference between the waterborne topcoat with 15% fluorane microcapsules on the basswood surface and the waterborne topcoat without microcapsules on the basswood surface. The color difference between the topcoat with and without microcapsules grew by 9.0 and 23.8, respectively, after the topcoat was aged in an oven at 120 °C for 40 h. The color difference between the topcoats with and without microcapsules rose by 58.3 and 37.6 in the oven at 160 °C for 40 h, respectively. The color difference between the topcoat with and without 15% fluorane microcapsules rose by 92.0 and 7.6, respectively, after 200 h in the UV climatic resistance test chamber.

The color difference of the topcoat increased with time for the same sample in the same aging condition. In the same environment and the same aging time, the color difference of coatings with 15% fluorane microcapsules was more obvious than those without fluorane microcapsules. The results show that after artificial accelerated aging, the topcoat with 15% fluorane microcapsules was more susceptible and in an unstable state, while the topcoat without fluorane microcapsules had a large degree of damage discoloration and bubble cracking, but it was still not obvious. This may be because the fluorane microcapsules in the topcoat had a high degree of degradation and discoloration after the aging test [40,41].

The effects of the topcoat aging time on gloss changes are shown in Table 11 for three incident angles of 20°, 60°, and 85°. The 60° gloss is taken as an example for analysis. The gloss of the topcoat without fluorane microcapsules reduced from 33.2% to 27.9% after being baked at 120 °C for 40 h, while the gloss of the topcoat containing 15% fluorane microcapsules decreased from 3.9% to 2.9%. The gloss of the topcoat without fluorane microcapsules reduced from 33.2% to 24.8% after being baked at 160 °C for 40 h, while the gloss of the topcoat with 15% fluorane microcapsules decreased from 3.9% to 2.7%. The gloss of the topcoat without fluorane microcapsules declined from 33.2% to 23.1% in the UV climate resistance test chamber for 200 h, while the gloss of the topcoat with 15% fluorane microcapsules decreased from 3.9% to 3.2%.

The findings indicate that the gloss of the topcoat was inversely related to the length of time it took to age under the same content. In comparison to the gloss of the topcoat with 15% fluorane microcapsules, the gloss of the topcoat without the fluorane microcapsules was more noticeable as it aged. This is due to the fact that the gloss was decreased and the particles on the topcoat’s surface were smooth and compact after the addition of fluorane microcapsules [42,43]. The addition of fluorane microcapsules significantly increased the gloss of the coating, while the absence of these microcapsules caused significant gloss loss and created microcracks of varied sizes or even bubble breaking.

**Figure 8:**
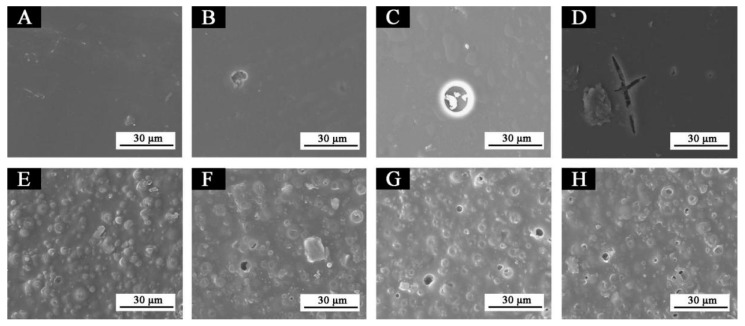
SEM images of sample 5# and sample 8# coatings before and after aging in different aging environments: (**A**) sample 5# before aging, (**B**) 120 °C [22], (**C**) 160 °C, (**D**) UV, (**E**) sample 8# before aging, (**F**) 120 °C, (**G**) 160 °C, (**H**) UV.

The SEM images of the topcoats without fluorane microcapsules and the topcoats with 15% fluorane microcapsules before and after aging experiments in the oven temperature of 120 °C, 160 °C, and UV climate resistance test chamber are shown in Figure 8. As can be seen from this figure, on the coating without fluorane microcapsules, large bubbles began to appear at 120 °C aging conditions and burst directly at 160 °C. Microcracks appeared after the topcoat was aged by the UV climate resistance test chamber, indicating that the topcoat was very obviously destroyed. However, the topcoat with 15% fluorane microcapsules showed tiny bubbles in the three aging environments, and the damaged area of the topcoat was reduced, compared to that without microcapsules.

## 4. Conclusions

The color of the coating with fluorane microcapsules can be reversibly changed, and the thermochromic temperature range is 30–32 °C. The fluorane microcapsule content significantly affected the color difference of the topcoat. The activation temperature of topcoats with microcapsule contents of 5–30% is 32 °C. The gloss of the topcoat is higher when the microcapsule is 5–15%, which is 8.3–11.9%. The waterborne topcoats with fluorane microcapsules have better mechanical properties if the content of fluorane is not more than 20%. The adhesion was grade 0–1, the hardness was up to 2H–3H, the impact resistance was up to 11.0 kg·cm, and the elongation at break was 0.89–23.46%. The red ink resistance of the topcoats was poorer. The artificial accelerated aging experiments showed that, with the same content, the color difference of the topcoat increased, and the gloss decreased gradually as the aging time increased. Overall, the performance of the waterborne topcoat with 15% fluorane microcapsules on Basswood is good, with a color difference of 71.9 at 32 °C, a gloss of 3.9% at 60°, an adhesion grade of 0, a hardness of 2H, an impact resistance of 10 kg·cm, and an elongation at break of 15.56%. In the same aging environment and the same aging time, the color difference of the topcoat with 15% fluorane microcapsules was more obvious. The damaged area of the topcoat with the addition of 15% fluorane microcapsules was reduced and the coating had a certain aging resistance. The research results provide a technical reference for the application of thermochromic microcapsules in topcoats for wood materials.

## Figures and Tables

**Table 1:** Arrangement of the orthogonal test.

Sample (#)	Fluorane Microcapsule Content (%)	Drying Temperature (°C)	Drying Time (h)
1	15	35	2
2	15	60	4
3	30	35	4
4	30	60	2

**Table 2:** The ingredients of waterborne thermochromic topcoats.

Sample (#)	Fluorane Microcapsule Content (%)
1	15
2	15
3	30
4	30
5	0
6	5
7	10
8	15
9	20
10	25
11	30

**Table 3:** Grade of liquid resistance of topcoats.

Grade	Situation
1	No observable change (no damage)
2	There is only tiny apparent darkening or discontinuous marks when the light hits the test surface or is very close to the mark and reflects to the observer’s eye.
3	A faint impression that may be seen from multiple angles, such as a nearly full circle or ring.
4	The surface structure changes little.
5	Severe impact, modification of the surface’s structure, complete or partial shredding of the surface material, or paper adherence to the test surface.

**Table 4:** Range analysis of the orthogonal test.

Sample	Fluorane Microcapsule Content (%)	Drying Temperature (°C)	Drying Time (h)	Color Difference Results during Heating Process (16–32 °C)
1	15	35.0	2.0	68.6
2	15	60.0	4.0	70.6
3	30	35.0	4.0	72.0
4	30	60.0	2.0	74.9
Mean 1	69.60	70.30	71.75	
Mean 2	73.45	72.75	71.30	
Range	3.85	2.45	0.45	

**Table 5:** Effect of fluorane microcapsule concentration on the gloss of topcoats.

Sample (#)	Fluorane Microcapsule Content (%)	20° Gloss (%)	60° Gloss (%)	85° Gloss (%)
5	0	8.0	33.2	52.9
6	5	1.9	5.0	11.9
7	10	1.8	4.5	8.3
8	15	1.9	3.9	10.7
9	20	1.6	2.7	6.8
10	25	1.8	2.7	7.9
11	30	1.8	2.4	8.7

**Table 6:** Effect of fluorane microcapsule concentration on mechanical properties.

Sample (#)	Fluorane Microcapsule Content (%)	Hardness (H)	Adhesion (Level)	Impact Resistance (kg∙cm)	Elongation at Break (%)
5	0	H	0	5	17.93
6	5	2H	0	8	22.73
7	10	2H	0	9	23.46
8	15	2H	0	10	15.56
9	20	3H	0	10	52.00
10	25	3H	1	11	3.78
11	30	3H	1	11	0.89

**Table 7:** The color difference of the topcoat with fluorane microcapsules after liquid resistance.

Sample (#)	Fluorane Microcapsule Content (%)	∆*E*_94_			
		Red Ink	NaCl	Ethyl Alcohol	Detergent
5	0	7.1	0.6	0.7	1.0
6	5	20.5	2.5	3.0	2.9
7	10	39.1	2.9	2.9	2.3
8	15	40.0	2.8	2.5	3.0
9	20	40.1	0.8	1.7	5.8
10	25	64.3	2.9	10	11.1
11	30	67.7	2.3	4.2	14.1

**Table 8:** The 60° gloss of the topcoat with fluorane microcapsules after liquid resistance.

Sample (#)	Fluorane Microcapsule Content (%)	Gloss after Red Ink (%)	Gloss after NaCl (%)	Gloss after Ethyl Alcohol (%)	Gloss after Detergent (%)
5	0	32.2	32	31.6	32.1
6	5	4.4	4.8	4.9	5.1
7	10	4.0	4.0	4.2	4.6
8	15	3.4	4.1	4.4	4.2
9	20	2.4	2.7	2.7	2.7
10	25	2.4	2.6	2.6	3.0
11	30	2.1	2.3	2.3	2.7

**Table 9:** The resistance to liquid level of the topcoat with fluorane microcapsules.

Sample (#)	Fluorane Microcapsule Content (%)	Level after Red Ink (Grade)	Level after NaCl (Grade)	Level after Ethyl Alcohol (Grade)	Level after Detergent (Grade)
5	0	2	1	1	1
6	5	3	1	1	1
7	10	3	1	1	1
8	15	3	1	1	1
9	20	4	1	1	2
10	25	4	1	1	2
11	30	4	1	1	2

**Table 10:** Effect of aging time on the color difference of the topcoat.

Aging Environment	Fluorane Microcapsule Content (%)	Ageing Time (h)	ΔL*	Δa*	Δb*	Δ*E*_94_
Oven to 120 °C	0	0	0	0	0	0
		8	−4.5	1.5	1.0	4.8
		16	−4.2	2.4	1.7	5.1
		24	−3.9	2.5	2.7	5.4
		32	1.4	2.0	6.5	6.9
		40	3.2	0.4	8.4	9.0
	15	0	0	0	0	0
		8	−0.1	1.1	6.5	6.6
		16	−1.5	−1.1	13.3	13.4
		24	−0.5	−2.2	14.8	15
		32	0.3	1.1	18.5	18.5
		40	0.2	5.5	23.2	23.8
Oven to 160 °C	0	0	0	0	0	0
		8	11.1	−5.1	−20.5	23.9
		16	22.1	−12.0	−18.6	31.3
		24	24.7	−12.9	−17.3	32.8
		32	29.0	−14.9	−13.2	35.2
		40	30.3	−16.4	−15.0	37.6
	15	0	0	0	0	0
		8	7.9	1.2	20.4	21.9
		16	13.2	2.8	33.2	35.8
		24	19.8	2.9	47.2	51.3
		32	19.5	2.2	49.2	53.0
		40	22.2	2.4	53.9	58.3
UV	0	0	0	0	0	0
		40	−0.9	5.3	3.9	6.6
		80	−2.9	4.9	3.7	6.8
		120	−3.9	4.1	4.5	7.2
		160	−4.2	5.2	3.4	7.5
		200	2.8	2.5	6.6	7.6
	15	0	0	0	0	0
		40	5.9	13.4	80.5	81.8
		80	6.1	11.8	79.1	80.2
		120	5.3	11.9	87.7	88.7
		160	4.4	12.4	88.7	89.7
		200	4.9	11.1	91.2	92.0

**Table 11:** Effect of aging time on 60° gloss of the topcoat.

Aging Environment	Fluorane Microcapsule Content (%)	Ageing Time (h)	20° (%)	60° (%)	85° (%)
Oven to 120 °C	0	0	8.0	33.2	52.9
		8	8.2	33.1	20.4
		16	8.0	32.6	21.2
		24	7.7	32.0	22.2
		32	7.6	30.4	20.2
		40	7.4	27.9	23.0
	15	0	1.9	3.9	10.7
		8	1.5	3.3	3.6
		16	1.6	3.2	3.5
		24	1.6	3.3	1.6
		32	1.5	3.0	3.1
		40	1.5	2.9	3.5
Oven to 160 °C	0	0	8.0	33.2	52.9
		8	7.9	32.3	34.1
		16	7.7	31.6	26.0
		24	7.8	29.0	22.0
		32	5.8	27.3	19.8
		40	5.7	24.8	20.7
	15	0	1.9	3.9	10.7
		8	1.0	2.9	3.8
		16	1.0	2.9	3.9
		24	0.9	2.9	4.5
		32	0.8	2.8	4.2
		40	0.7	2.7	4.3
UV	0	0	8.0	33.2	52.9
		40	7.6	32.6	45.7
		80	7.6	30.8	31.4
		120	6.9	28.6	29.2
		160	6.3	26.0	23.0
		200	5.1	23.1	19.1
	15	0	1.9	3.9	10.7
		40	1.7	3.6	3.6
		80	1.7	3.6	3.8
		120	1.6	3.5	3.5
		160	1.6	3.3	2.9
		200	1.7	3.2	3.9

## Data Availability

The data presented in this study are available on request from the corresponding author.
